# Generation of 3D Spheroids Using a Thiol–Acrylate Hydrogel Scaffold to Study Endocrine Response in ER^+^ Breast Cancer

**DOI:** 10.1021/acsbiomaterials.2c00491

**Published:** 2022-08-24

**Authors:** Anowar H. Khan, Sophia P. Zhou, Margaret Moe, Braulio A. Ortega Quesada, Khashayar R. Bajgiran, Haley R. Lassiter, James A. Dorman, Elizabeth C. Martin, John A. Pojman, Adam T. Melvin

**Affiliations:** † Department of Chemistry, Louisiana State University, Baton Rouge, Louisiana 70803, United States; ‡ Department of Bioengineering, Rice University, Houston, Texas 77005, United States; § Cain Department of Chemical Engineering, Louisiana State University, Baton Rouge, Louisiana 70803, United States; ∥ Biological and Agricultural Engineering, Louisiana State University, Baton Rouge, Louisiana 70803, United States

**Keywords:** tumor spheroid, microfluidics, droplet trapping array, hydrogel, ER^+^ breast cancer, endocrine therapy, fulvestrant, drug testing

## Abstract

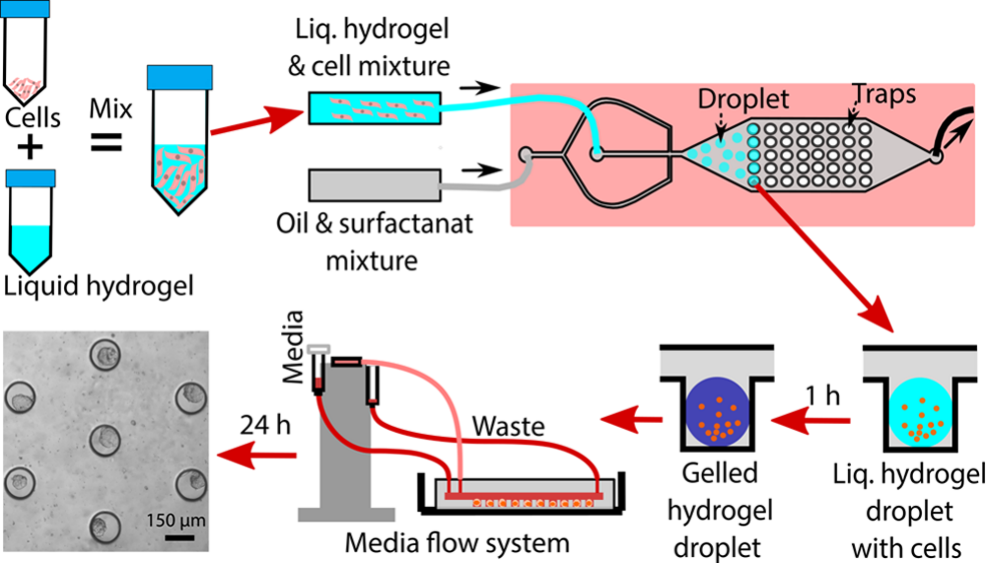

Culturing cancer cells in a three-dimensional (3D) environment better recapitulates *in vivo* conditions by mimicking cell-to-cell interactions and mass transfer limitations of metabolites, oxygen, and drugs. Recent drug studies have suggested that a high rate of preclinical and clinical failures results from mass transfer limitations associated with drug entry into solid tumors that 2D model systems cannot predict. Droplet microfluidic devices offer a promising alternative to grow 3D spheroids from a small number of cells to reduce intratumor heterogeneity, which is lacking in other approaches. Spheroids were generated by encapsulating cells in novel thiol–acrylate (TA) hydrogel scaffold droplets followed by on-chip isolation of single droplets in a 990- or 450-member trapping array. The TA hydrogel rapidly (∼35 min) polymerized on-chip to provide an initial scaffold to support spheroid development followed by a time-dependent degradation. Two trapping arrays were fabricated with 150 or 300 μm diameter traps to investigate the effect of droplet size and cell seeding density on spheroid formation and growth. Both trapping arrays were capable of ∼99% droplet trapping efficiency with ∼90% and 55% cellular encapsulation in trapping arrays containing 300 and 150 μm traps, respectively. The oil phase was replaced with media ∼1 h after droplet trapping to initiate long-term spheroid culturing. The growth and viability of MCF-7 3D spheroids were confirmed for 7 days under continuous media flow using a customized gravity-driven system to eliminate the need for syringe pumps. It was found that a minimum of 10 or more encapsulated cells are needed to generate a growing spheroid while fewer than 10 parent cells produced stagnant 3D spheroids. As a proof of concept, a drug susceptibility study was performed treating the spheroids with fulvestrant followed by interrogating the spheroids for proliferation in the presence of estrogen. Following fulvestrant exposure, the spheroids showed significantly less proliferation in the presence of estrogen, confirming drug efficacy.

## Introduction

It is estimated that 70% of newly diagnosed cases of breast cancer will be estrogen receptor positive (ER^+^).^1^ Despite the common receptor designation, there is significant intertumor heterogeneity among ER^+^ tumors, which results in differences in patient response to targeted endocrine therapies.^2^ Specifically, roughly 40% of ER^+^ breast cancers will have *de novo* resistance or develop resistance to endocrine therapy.^3,4^ Resistance to endocrine therapy typically correlates with a more poor prognosis in ER^+^ breast cancer patients along with increased cellular proliferation.^3,5^ One challenge associated with studying drug resistance in ER^+^ positive breast cancer is that the majority of models rely on two-dimensional (2D) cell culture systems, which cannot account for cell-to-cell or cell-to-matrix interactions that drive drug resistance and tumor proliferation.^6−10^ Moreover, many anticancer drugs that have been found to be effective against ER^+^ breast cancer in the 2D models dramatically fail during *in vivo* drug trials due to differences in drug sensitivity and cell biology found in three-dimensional (3D) models.^11,12^ 2D models have been established to be poor representations of *in vivo* conditions because they cannot account for the aforementioned cellular interactions or the mass transfer limitations of nutrients and drugs into the tumor and the cellular waste out of the tumor, resulting in a poor model for preclinical drug screening.^6,13−15^*In vitro* 3D tumor models better recapitulate the tumor microenvironment (TME) by mimicking strong cell-to-cell interactions and mass transfer limitations of metabolites, oxygen, and drugs overcoming several limitations associated with 2D models. Several approaches exist to generate 3D cell culture (herein referred to as 3D spheroids) including pellet culture, hanging droplet arrays, spinner flasks, liquid overlay, and magnetic levitation.^16−22^ While these approaches can successfully generate 3D spheroids, they suffer from several limitations. One of the major limitations of these techniques is weak cell-to-cell interactions resulting in spheroids that poorly mimic *in vivo* 3D TME.^9,23^ Furthermore, pellet culture lacks throughput and generates 3D spheroids that are vulnerable to shear stress from centrifugation.^19^ 3D spheroids generated by spinner culture, magnetic levitation, or liquid overlay techniques exhibit significant heterogeneity in both size and shape.^19,20^ Additionally, magnetic levitation is expensive with an overall low throughput coupled with the fact that the magnetic beads can be toxic at a higher concentration.^8,20^ Similarly, 3D spheroids generated in hanging drop arrays are difficult to visualize after aggregation, which limits real-time imaging.^8,21^ Recently, microfluidic devices have become a popular alternative to rapidly generate 3D spheroids. One common approach utilizes microwell arrays that have been modified to prevent cellular attachment and force cellular aggregation into 3D spheroids.^15,24−27^ These devices can rapidly generate a large number of spheroids; however, the spheroids suffer from rapid disaggregation and significant genetic heterogeneity since this technique generated spheroids by aggregating a large number of cells (>1000 cells).^24−28^ While it is important to study cellular heterogeneity across a population of spheroids (e.g., intertumor heterogeneity), the microwell approach can potentially misrepresent the 3D cellular response to drugs since spheroids generated from this technique contain significant intratumor genetic heterogeneity.^29^ An alternative high-throughput approach to generate 3D spheroids uses droplet microfluidics, which can precisely control the volume and cell density of the aqueous droplet by varying the flow rate of the oil and aqueous phase, resulting in size-controlled spheroids in a high throughput manner that can be imaged in real-time.^30,31^ Droplet microfluidics also allows for precise spheroid recovery from a particular trap with appropriate settings, which many conventional systems lack.^30^ Furthermore, droplet microfluidic trapping arrays, which are capable of encapsulating ∼1–10 cells in aqueous droplets, offer promise to grow, interrogate, and study >400 individual spheroids in a single device, which can overcome the substantial intratumor heterogeneity observed *via* the forced aggregation approach.

Another challenge in the generation of 3D spheroids is to mimic the tumor extracellular matrix (ECM), an acellular network of extracellular macromolecule including proteins and polysaccharides that drive cellular proliferation.^32,33^ The majority of the aforementioned techniques suspend the cells in growth media and then rely on either gravitational or centrifugal force to generate spheroids without any physical scaffold to support cell growth or generation of the tumor ECM.^15,21,24,34−36^ Recent studies suggest that, in the absence of ECM, 3D spheroids exhibit poorer cell-to-cell communication and are physiologically less relevant compared to spheroids generated in ECM-containing systems.^9,23,37^ The droplet-based approach overcomes this limitation by incorporating different types of soft hydrogel materials that can serve as a scaffold for 3D cell culture. Several studies have demonstrated that a hydrogel scaffold provides mechanical forces for cancer cells to support spheroids possessing strong cell-to-cell interactions, extracellular matrix deposition between cells, and gradients in nutrient concentration from the core to the shell of the spheroid.^9,32,38^ There are currently few hydrogels being used to generate 3D spheroids including agarose, alginate, matrigel, collagen, or polyethylene glycol (PEG)-based hydrogels.^30,32,39−41^ While these hydrogels facilitate the spheroid generation, they have a few underlying weaknesses including both gelation time and degradation of the hydrogel. For example, the temperature of the device needs to be increased to degrade an agarose hydrogel to recover the spheroids, which can bias the cellular response.^30^ Similarly, alginate requires harsh chemical treatment to degrade the hydrogel coupled with the need for excess calcium at the beginning of experimentation to facilitate gelation, both of which can bias the cellular response.^32,41^ Matrigel and collagen are expensive, difficult to work, suffer from substantial batch-to-batch heterogeneity, and are difficult to incorporate into microfluidic devices.^41^ In an effort to overcome these limitations, the current work aims to incorporate a novel thiol–acrylate (TA) hydrogel scaffold that supports initial 3D spheroid formation, which starts to degrade after ∼24 h, allowing for the generation of highly stable, uniform 3D spheroids. Prior work by Khan et al. described the development of a synthetic TA hydrogel that allows for precise control of the gelation time and degradation by varying the weight percentage of the polymers present in the hydrogel and the pH of the hydrogel.^42−45^ Moreover, the TA hydrogel is biodegradable in culture media within 48 h of gelation and requires no additional chemical treatment to degrade the scaffold, which makes it very useful for further spheroid analysis.^42^ The TA hydrogel was demonstrated to support 3D cell growth for >10 days in two model breast cancer cell lines including a model ER^+^ MCF-7 cell line.

The goal of this study is to utilize a two-layer polydimethylsiloxane (PDMS)-based microfluidic droplet trapping array incorporating the TA hydrogel scaffold to generate and interrogate ER^+^ 3D spheroids. The device utilizes a continuous gravity-driven media infusion system to supply the 3D spheroids with fresh media to avoid growth stagnation, which has been demonstrated previously.^17,30,46,47^ Two different-sized droplets (and traps) were investigated (150 and 300 μm diameters) to validate the ability to generate different-sized viable 3D spheroids. A custom MATLAB code was developed to measure spheroid size using brightfield microscopy to decrease analysis time and increase overall throughput. As part of this study, it was identified that a minimum of 10 encapsulated MCF-7 cells were needed to successfully generate a 3D spheroid, providing new insight into the minimum number of cells needed to support 3D cell culture. To validate the utility of the system in ER^+^ breast cancer, a drug susceptibility study was performed, treating the spheroids with fulvestrant (an estrogen-targeted therapeutic, ICI-182780) followed by interrogating the spheroids for proliferation in the presence of 17β-estradiol (E2 or estrogen). The MCF-7 cells were still found to proliferate when exposed to an intermediate dose (50 nM) of fulvestrant in the presence of estrogen, requiring a significantly higher dose (100 nM) to prominently reduce proliferation. However, this intermediate dose for the 3D system is still substantially higher than the concentration of fulvestrant required to alter endocrine signaling in the 2D culture system, which supports the concept of an altered endocrine response in the 3D tumor environment when compared to 2D systems.

## Materials and Methods

### Microfluidic Device Fabrication

Two different microfluidic droplet trapping arrays were used in this study incorporating different sized traps (150 and 300 μm diameters). The device with 150 μm traps had an array of 990 traps (herein called the 150 μm trapping array), while the one with 300 μm traps had an array of 450 traps (herein called the 300 μm trapping array). Both devices were fabricated by soft lithography (see the Supporting Information). The devices consist of two polydimethylsiloxane (PDMS) layers: the first layer contains the fluidic channel and the trapping array connected with the fluidic layer. To make a 2 mm thick PDMS device replica, the base and curing agent were mixed in a ratio of 10:1 and degassed under a vacuum for 45 min before pouring onto the silicon master. The second layer is a flat 3 mm thick PDMS layer with no channels that functions as the top layer of the device consisting of 20 g of degassed PDMS mixture with the same ratio of base and curing agent poured into a 100 mm Petri dish and placed on a hot plate at 65 °C for 12 h to cure completely. The PDMS device replica and flat layer were carefully removed from the wafer and Petri dish and cut to size with an X-Acto knife. The flat layer and device replica were aligned visually to punch holes in the flat layer side at two inlets and one outlet using a blunted 18-gauge needle. The flat layer and device replica were bound together *via* treatment with oxygen plasma for 1 min. The surface inside the device was made hydrophobic by treatment with Aquapel for ∼30 s followed by removal by flowing high-pressure nitrogen gas and then Novec 7500 oil through the device. Six 14” long sections of Tygon tubing (0.022” inner diameter × 0.042” outside diameter, Cole-Parmer) were cut and autoclaved to ensure sterility during experimentation.

### Cell Culture

MCF-7 cells (ATCC) were maintained with DMEM (Corning) supplemented with 10% v/v HyClone cosmic calf serum (VWR Life Sciences Seradigm), 1% MEM essential amino acids (Quality Biological Inc.), 1% MEM nonessential amino acids (Quality Biological Inc.), 1 mM sodium pyruvate (Thermo Fisher Scientific), and 48 ng insulin/mL media (Insulin, human recombinant dry powder, Sigma-Aldrich). Cells were maintained in T-75 flasks in a humidified incubator at 37 °C and 5% v/v CO_2_. Cells were subcultured when they reached ∼80% confluency by first being washed with 1X phosphate-buffered saline (PBS: 137 mM NaCl, 10 mM Na_2_HPO_4_, 27 mM KCl, and 1.75 mM KH_2_PO_4_ at pH 7.4) containing 3.7 mM EDTA (Corning) for 2 min and followed by 7 min incubation at 37 °C before being reseeded into a new T-75 flask.

### Generation and Trapping of MCF-7-Laden TA Hydrogel Droplets

The droplet generator used in this system has two inlets, where one was used to inject Novec 7500 oil containing 0.5% (w/w) fluorosurfactant mixture, and the other inlet was used to inject the unpolymerized TA hydrogel containing the MCF-7 cells. Prior to experimentation, a 5 mL syringe containing only Novec 7500 was connected to aqueous inlet to remove all of the air from the device. Next, Tygon tubing was connected from the oil inlet to a 5 mL syringe containing Novec 7500 oil with 0.5% (w/w) fluorosurfactant. Afterward, the TA hydrogel/MCF-7 suspension was prepared. First, 8.5 wt % TA hydrogel was made in extracellular buffer (ECB; pH 7.7) as previously reported by Khan et al.^42,45^ In brief, 9 μL of NaOH (2M) was added to 5 g of ECB to make the reaction medium basic enough for thiol to react with acrylate groups. Next, polyethylene glycol diacrylate (0.2020 g, PEGDA 700) was added, and the suspension was vortexed for ∼10 s. Finally, a three-arm thiol (0.2626 g, ETTMP 1300) was added to the reaction mixture and vortexed vigorously for ∼30 s. MCF-7 cells were detached from the culture flask and spun at 300x g for 6 min to obtain a concentrated cell pellet. This cell plate was then resuspended with 500 μL of hydrogel precursor solution to achieve a final concentration of 8 × 10^6^ cells/mL hydrogel for the 150 μm trapping array or 5 × 10^6^ cells/mL hydrogel for the 300 μm trapping array device (Table S1). The TA hydrogel/MCF-7 suspension was transferred to a 1 mL syringe connected to 23-gauge needle, which was connected to the gel inlet of the device using Tygon tubing. Once all the syringes were connected to the device, flow was initiated (see Table S1 for flow rate details) using two Harvard syringe pumps. After all the traps were filled with droplets and extra droplets started to flow through the outlet tubing, droplet generation was halted by stopping flow from the TA hydrogel/MCF-7 syringe while still flowing the oil/surfactant syringe for 5 min at a rate of 1000 μL/h. Followed by the removal of extra droplets, the oil/surfactant syringe was swapped with a syringe containing only Novec 7500 oil, which was flushed through the device for 60 min at a rate of 1000 μL/h to remove any residual fluorosurfactant trapped inside the device. During this 60 min wait period, the entire device was imaged using a Leica DMi8 inverted microscope outfitted with brightfield applications at 5× objective for day 0. Once hydrogel droplets were polymerized, the oil syringe was swapped with a 3 mL syringe containing complete growth media, which was infused into the device for 10 min at a rate of 1000 μL/h. The device was then connected with a home-built gravity-driven media flow system and incubated at 37 °C for the duration of the experiment. Finally, brightfield images of the entire microfluidic device were taken over the span of a week to monitor spheroid growth and morphology changes.

### Viability Assay

To determine spheroid viability throughout the culture period, live and dead staining was carried out after 7 days of on-chip cell culture using the live stain Calcium AM (Life Technologies), the dead stain Ethidium homodimer-1 (EthD-1, Life Technologies), and nuclei stain Hoechst 33342 of concentrations 3.75, 5, and 60 μM, respectively, made in ECB. Live, dead, and nuclei stain were flown through the device at a rate of 350 μL/h for 4 h using a Harvard syringe pump at 37 °C. Cellular fluorescence was visualized using a Leica DMi8 inverted microscope outfitted with a FITC filter cube, rhodamine filter, DAPI filter, and brightfield applications at 10× objective. Digital images were acquired using a Flash 4.0 high-speed camera (Hamamatsu) with a fixed exposure time of 15 ms for the FITC filter (green, live cells), 100 ms for the rhodamine filter (red, dead cells), 35 ms for DAPI (blue, nucleus), and 25 ms for brightfield.

### Drug Response Assay

The initial stock of fulvestrant (ICI-182780) and estrogen was made and serially diluted in DMSO and kept at −20 °C until further use. Stripped media (phenol free DMEM media containing 5% FBS charcoal dextran, 1% glutamax, 1% nonessential amino acid, 1% essential amino acid, 1% sodium pyruvate, and 1% penicillin–streptomycin) aliquots were generated with either fulvestrant (50 and 100 nM) or estrogen (100 pM) and stored at 4 °C. To study the effect of fulvestrant or estrogen on spheroid growth, spheroids were grown in the 300 μm trapping array for 72 h in cell culture media, followed by 24 h of culture in stripped media. For the drug study, the spheroids were cultured in the fulvestrant spiked stripped media for 9 h by flowing 4 mL of media through the device. Afterward, the fulvestrant spiked media was removed from the reservoir and waste collector and replaced with 4 mL of 17β-estradiol (E2 or estrogen) spiked stripped media. The estrogen-spiked media was replenished every 24 h. A vehicle control experiment was performed the same using DMSO (2 μL of DMSO in stripped media) in lieu of fulvestrant. Terminal Proliferation (*K*_i_-67, Bio Legend) and nuclei (Hoechst 33342, Thermo Scientific) staining were carried out as described below.

### Immunofluorescent Staining and Imaging

At the end of experiment, the spheroids were fixed by flowing 4% paraformaldehyde (PFA) solution using a syringe pump (600 uL/h) for 2 h. Following a wash with PBS for 30 min at a rate of 600 μL/h, the samples were then permeabilized by flowing PBS containing 1% (w/v) Triton X 100 overnight using gravity-driven flow. On the next day, the spheroids were washed with a blocking buffer (0.5% w/v BSA in PBS) for 1 h using a syringe pump followed by a PBS wash for 30 min. Stain solution was made under a biosafety hood containing 490 μL of 0.25% BSA, 7 μL of *K*_i_-67 stain (1:70 dilution), and 3 μL of nuclear stain Hoechst 33342 (60 μM). Afterward, the stain solution was transferred into a 1 mL syringe and flown through the device overnight at a rate of 70 μL/h using a syringe pump at room temperature in the dark. Samples were washed for a final time with PBS for 30 min using a gravity-driven system prior to imaging. Cellular fluorescence was visualized using a Leica DMi8 inverted microscope outfitted with a FITC filter cube (excitation 460–500 nm, emission 512–542 nm), DAPI filter (excitation 325–375 nm, emission 435–485 nm), and brightfield applications at 20× objective. Digital images were acquired using a Flash 4.0 high-speed camera (Hamamatsu) with a fixed exposure time of 600 ms for the FITC filter (green, *K*_i_-67 positive cells), 50 ms for DAPI (blue, nucleus), and 35 ms for brightfield.

### Statistical Analysis

Experiments with replicate data were represented as arithmetic mean ± standard deviation. Statistical differences between different groups were determined by standard one-way ANOVA and Fisher LSD test using Origin software. If the *p*-value <0.001 then data sets were considered as statistically significant (***) while a *p*-value >0.05 was considered statistically nonsignificant (ns).

## Results and Discussion

### Generation and Isolation of ER^+^ Breast Cancer 3D Spheroids Using a TA Hydrogel Scaffold

**Figure 1:**
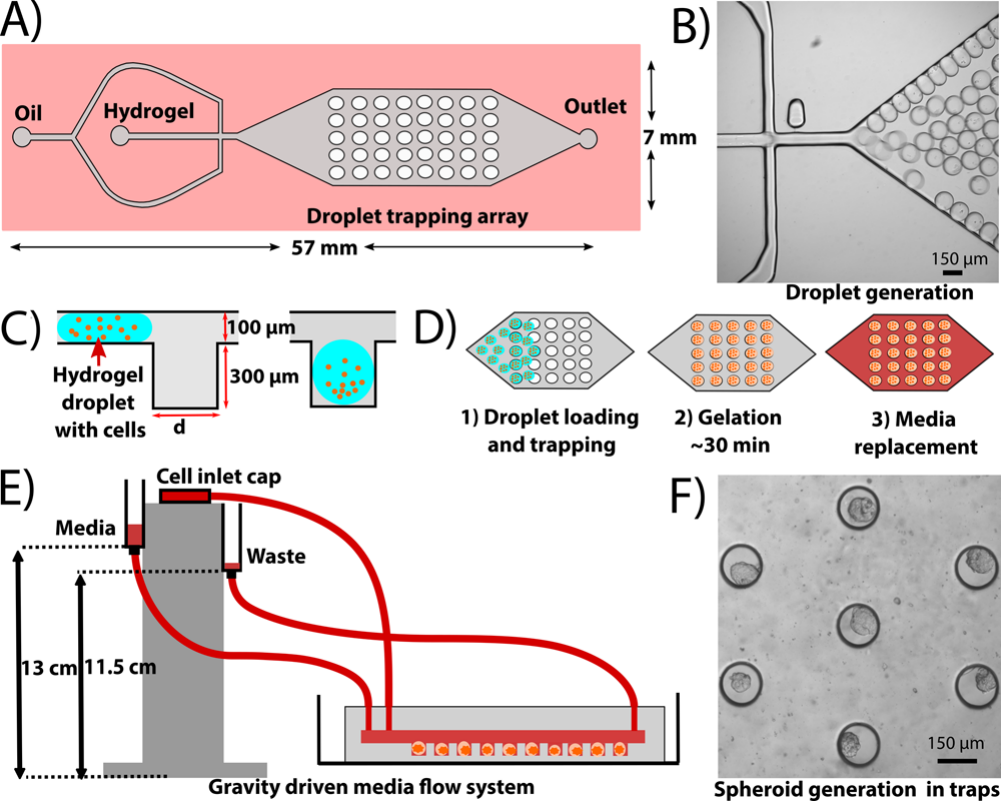
Generation of 3D spheroids using a microfluidic droplet trapping array and thiol–acrylate hydrogel scaffold. (A) Top view of the droplet trapping array showing two inlets for carrying oil and aqueous hydrogel, a flow-focusing junction, the droplet trapping array, and a single outlet. (B) Hydrogel droplet generation at the flow-focusing junction. (C) Side view of the flowing droplet through the fluidic channel to isolate into the traps. Heights of the fluidic layer and traps are 100 and 300 μm, respectively, for both the 150 and 300 μm trapping arrays. (D) Protocol of spheroid generation begins with droplet loading and trapping; later, media replenishes oil after the gelation of the hydrogel droplets. (E) Schematic representation of gravity-driven media flow setup for the microfluidic droplet generator. (F) Brightfield image of MCF-7 spheroids on day 7 in the 150 μm trapping array.

The microfluidic droplet trapping arrays consist of two components: an upstream flow-focusing junction to generate the cell-laden hydrogel droplets and a downstream underneath circular trapping array to isolate and culture the 3D spheroids (Figure 1A). Droplet generation occurs at the flow focusing T-junction, which consists of a 50 μm wide oil carrying channel and an 88 μm wide aqueous gel carrying channel (Figure 1B, Movie S1). The average size of the droplets was found to be 153 ± 16 and 311 ± 24 μm generated in 150 and 300 μm trapping arrays, respectively, at the specific flow rate mentioned in Table S1. The cell-laden droplets were then carried through the oil phase and settled in the underneath circular trapping array (Figure 1C, Movie S2). An aqueous 8.5 wt % thiol–acrylate (TA) hydrogel was used as a scaffold material for MCF-7 cells since the presence of scaffold better recapitulates *in vivo* conditions.^32,42^ TA hydrogel gelation occurs rapidly (∼35 min) due to a base-catalyzed Michael addition reaction allowing for sufficient time to generate and trap the droplets prior to scaffold polymerization and ultimate media replacement to support 3D cell growth (Figure 1D, Movie S3). Both 150 and 300 μm trapping arrays were capable of ∼99% droplet trapping with the 300 μm droplets yielding ∼90% cellular encapsulation and the 150 μm droplets yielding ∼55% cellular encapsulation. A population of ∼500 cell-laden droplets exhibited a range of 5–25 cells/droplet in the 150 μm trapping array and 10–45 cells/droplet in the 300 μm trapping array. The higher cell trapping efficiency and higher number of cells per droplet for the 300 μm trapping array were due to the fact that the larger diameter traps could hold a larger volume of gel, which increased the probability of trapping cell-laden droplets, which has been previously shown in the literature.^48^ The microfluidic system was found to generate spheroids faster than other comparable techniques like microplates, hanging drop array, magnetic levitation, or spinner flask where it can take anywhere from 7 to 14 days to generate spheroids.^13,49−51^ The microfluidic approach described here was capable of generating spheroids within 24 h of trapping the cells in the array. Furthermore, spheroids were seen to form around the same time (within the first 24 h of trapping) regardless of the number of isolated cells in each trap as long as each trap contain more than 10 cells. The 300 μm trapping array generated 330–360 spheroids in a single device, while the 150 μm trapping array generated 400–450 spheroids in a single device. This is a significantly higher number of generated spheroids when compared to similar systems.^15,26,52,53^

One challenge associated with on-chip growth of spheroids is the need to continually supply the cells with growth media.^15,26,39^ Several studies have shown growth stagnation after ∼72 h without media replenishment, motivating the need to continually infuse the system with fresh media.^15,39^ This can be done using syringe pumps; however, they are expensive, difficult to move around, and have a large footprint in the incubator, which can limit the number of devices that can be run in parallel. Similarly, the exchanging of syringes has the possibility of introducing air bubbles into the device, which can compromise the experiment. Instead, a gravity-driven media infusion system was used to avoid these complications (Figure 1E). Gravity-driven media infusion systems have previously been reported in the literature as an ideal alternative to pressure-driven flow to supply cells with sufficient media.^26^ The gravity-driven system consisted of two 5 mL syringes connected to the inlet and outlet ports of the device where the media infusion syringe was positioned slightly higher (∼2 cm) to induce flow due to the hydrostatic pressure (Figure S1). For cell culture purposes, media was replenished every 24 h by adding 4 mL of culture media into the media reservoir and at the same time removing spent media from the waster collector (Figure 1E, Figure S1). COMSOL simulations were performed to ensure that the gravity-driven flow did not result in substantial fluid shear stress (FSS) on the trapped spheroids. The velocity inside the traps was found to be approximately 0 m/s, proving that the design is able to isolate the spheroids and prevent them from being exposed to FSS for the bottom of the traps (Figure S2).

The result was a self-contained system capable of growing 3D spheroids for up to 7 days (Figure 1F). In addition to providing the cells with culture media, the gravity-driven infusion system also allows for facile biological interrogation of the cells, including exposing the cells to drugs or other biomolecules to induce a response. This, coupled with the small incubator footprint, allows for 6–12 devices to be run in parallel for the high-throughput screening of 3D spheroids. Furthermore, spheroids generated using this system has stronger cell-to-cell interaction and compact packing morphology (Figure 1F). The generated spheroids were found to be highly stable with no visible disaggregation during continuous media infusion overcoming a challenge associated with scaffold-less systems with poor cell-to-cell interaction.^9,54^

### High-Throughput Generation of ER^+^ Breast Cancer Spheroids Coupled with Automated Image Analysis

**Figure 2:**
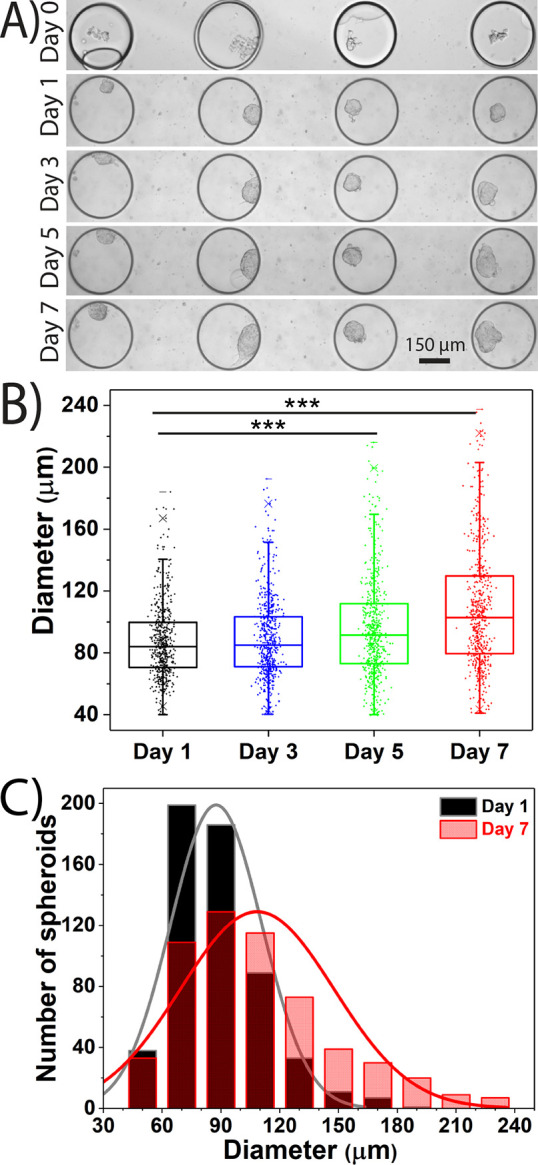
Generation of ER^+^ MCF-7 spheroids in the 300 μm microfluidic trapping array. (A) Brightfield images of four representative traps containing MCF-7 spheroids were collected at days 1, 3, 5, and 7. (B) Calculated diameters of a population of 700 spheroids accomplished using a custom MATLAB algorithm confirms cell growth in the device. (C) Size distribution of the generated spheroids on days 1 and 7 shows a shift in spheroid size as a function of time. ***p < 0.001.

Cellular growth was confirmed by culturing the cells and tracking the change in spheroid diameter and area for ∼700 spheroids generated inside the device for 7 days. Both the 150 and 300 μm trapping arrays were used to determine if droplet (and trap) size influenced cellular growth. The diameter and area of the generated spheroids were measured using a custom MATLAB image analysis code (see the Supporting Information) using a similar approach that automates data analysis for spheroid analysis that has been described in the literature with an unpublished source code.^30^ The code outlines and converts brightfield images into grayscale, filters out noise, masks the traps, and isolates the spheroids inside, and finally, the identified spheroids allow for exact calculation of the pixelated area occupied by each spheroid as well as circularity and diameter (Figure S3). This code allowed for rapid (<15 min) analysis of 125 images containing 2000–3900 traps (depending on the type of droplet trapping array being used in an experiment), which is superior to other existing image analysis algorithms.^55^ The diameters of all 700 spheroids were found to increase during the 7 day incubation in the 300 μm trapping array (Figure 2A,B) with similar results observed in the 150 μm trapping array (Figure S4A,B). The gradual increase in diameter of the spheroids over the 7 day period can be correlated to a greater number of cells confirming cell growth in both the 150 and 300 μm trapping arrays. A similar trend in both devices also confirms that starting (and ending) trap or droplet diameter does not impact cell growth. Day 7 data showed that the 300 μm trapping array generated spheroids with an average diameter is 108.5 ± 39.2 μm (Figure 2C), while the 150 μm trapping array generated spheroids with an average diameter of 62.6 ± 12.2 μm (Figure S4C). The day 7 size distribution of the spheroids was found to be much wider in the 300 μm trapping array when compared to the 150 μm trapping (Figure 2C, Figure S4C). This can be attributed to the fact that the range of trapped cells in the droplets was much wider in the 300 μm trapping array when compared to that of the 150 μm trapping array. The observed size distribution in both devices is still superior when compared to forced-aggregate culture systems, which exhibit a distribution of anywhere from 100 to 200 μm.^13,24,39,42,52,54^

**Figure 3:**
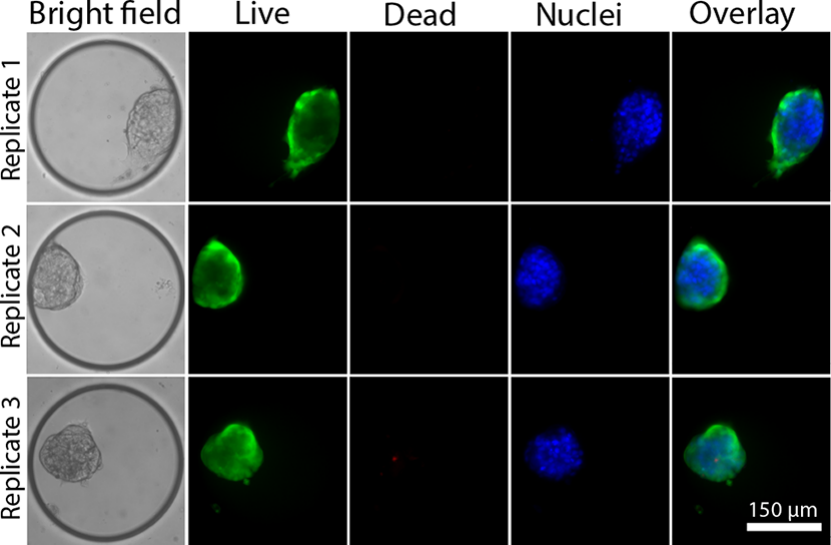
On-chip viability staining of MCF-7 spheroids generated in the 300 μm trapping array. After 7 days of culture, the spheroids were incubated with live and dead fluorescent stains. Representative images of three spheroids are shown for bright field, green (live, Calcein-AM), red (dead, ethidium homodimer), blue (nuclei, Hoechst 33342), and an overlay image.

Cellular viability was confirmed using a standard live–dead stain after 7 days of culture. Terminal staining indicated mostly viable spheroids in both the 300 μm (Figure 3) and 150 μm trapping arrays (Figure S5). Nuclei staining in both the 300 μm (Figure 3) and 150 μm trapping arrays (Figure S5) indicates close packing of the cells within the spheroids indicative of a compact distribution and strong cell-to-cell communication.^56^

### Minimum Number of Encapsulated Cells Is Required to Generate a Growing MCF-7 Spheroid

**Figure 4:**
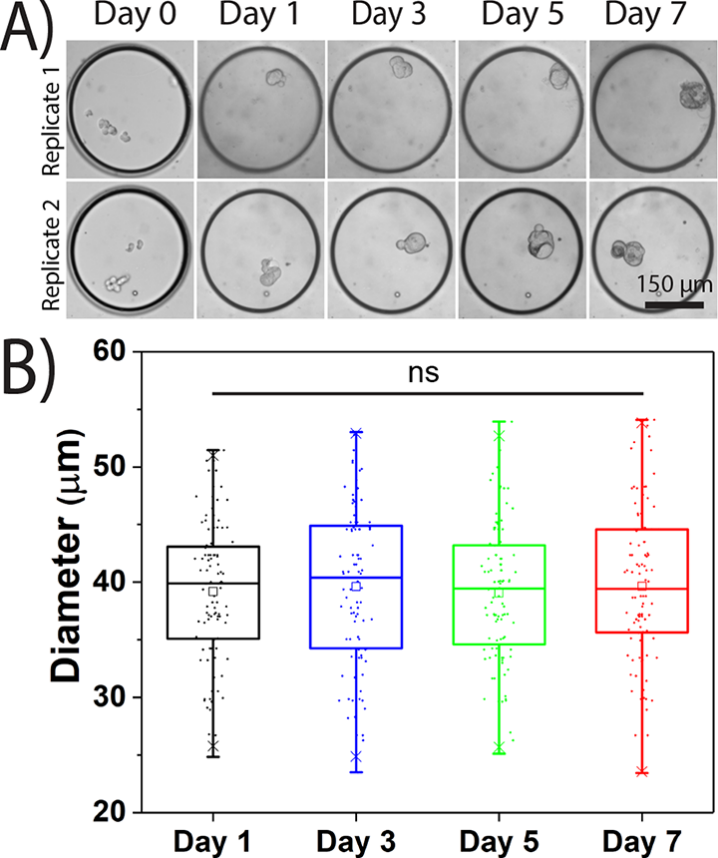
Fewer than 10 encapsulated MCF-7 cells resulted in poor spheroid growth in the 300 μm trapping array. (A) Brightfield images of traps containing less than 10 MCF-7 cells after encapsulation were taken at different time intervals. (B) Size of the 3D spheroids generated from less than 10 cells over time (ns indicates statistically nonsignificant when *p* > 0.05).

It is believed that cancer cells possess stem cell-like characteristics and they can repopulate from a small number of cells.^57,58^ Therefore, one of the goals of this work was to find out the minimum number of MCF-7 cells required to generate a spheroid that will grow over time. It is difficult and time-consuming for other common techniques such as spinner flask, well-plates, etc., since it is hard to control the size of the cell aggregate in these systems.^52^ However, a microfluidic droplet generator is one of the best choices for this type of study, since initial cells per droplet can be controlled by varying both cell density and droplet size. Experiments were carried out in both the 150 and 300 μm trapping arrays to determine the minimum number of cells required to generate a growing spheroid. To reduce the number of encapsulated cells, the cell density in the TA hydrogel syringe was decreased to 2.5 × 10^6^ cell/mL (for the 300 μm trapping array) and 4 × 10^6^ cell/mL (for the 150 μm trapping array), resulting in fewer than 10 encapsulated cells per droplet. Following a 7 day culture period (see Figure 1), the array was imaged to evaluate spheroid diameter and growth. Droplets containing fewer than 10 encapsulated cells produced stagnant 3D spheroids with no apparent change in diameter over the 7 day incubation period (Figure 4A). A one-way ANOVA indicated no significant change in spheroid diameter when comparing day 1 images to day 7 images (Figure 4B). This is in stark contrast to spheroids generated from droplets containing greater than 10 encapsulated cells, (Figure 2B), supporting the concept that a minimum number of cells is required to generate a growing spheroid. A similar trend was also seen in the 150 μm trapping array (Figure S6), which indicates that, regardless of droplet size, spheroid growth is strongly dependent on the number of encapsulated cells in the droplet when using the TA hydrogel scaffold.

### Evaluation of an Altered Response to Endocrine Therapy in ER^+^ MCF-7 3D Spheroids

**Figure 5:**
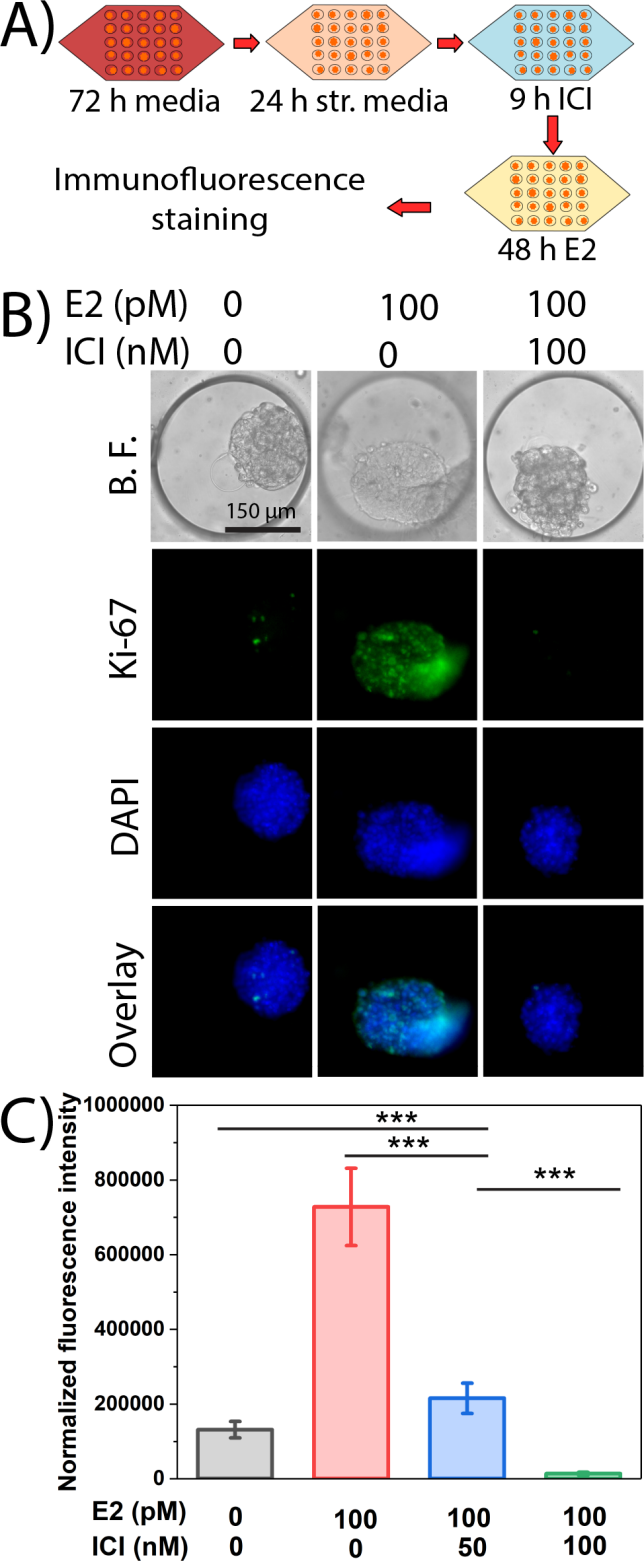
ER^+^ breast cancer exhibits an altered response to endocrine therapy in 3D cultured cells in the 300 μm trapping array. (A) Schematic of different steps of the drug resistance study. (B) Evaluation of cellular proliferation by *K*_i_-67 induced by estrogen (E2) in the presence or absence of fulvestrant (ICI) in MCF-7 spheroids. (C) Quantification of normalized fluorescence coupled with one-way ANOVA to demonstrate statistically significant changes in cellular proliferation due to drug treatments (*** indicates statistically significant *p* < 0.001, ns indicates statistically nonsignificant *p* > 0.05).

Changes in estrogen-mediated proliferation in the presence of the endocrine therapeutic fulvestrant (ICI-182780) in 3D cultured cells was studied to validate the utility and high throughput drug screening capabilities of the microfluidic system. The antiproliferative drug fulvestrant, which selectively degrades the estrogen receptor, was chosen because patients with ER^+^ breast cancer can benefit from selective estrogen receptor down-regulator (SERD) drug treatment because it possesses a higher antitumor activity than tamoxifen and fewer side effects.^36,59−61^ A drug susceptibility study was performed treating the spheroids with fulvestrant followed by interrogating the spheroids for proliferation in the presence of 17β-estradiol (E2 or estrogen). Briefly, 3D spheroids were grown for 72 h in the 300 μm trapping array and then starved in a stripped media for 24 h to nullify any exogenous estrogen stimulation. Then, spheroids were exposed to 50 and 100 nM fulvestrant for 9 h followed by 48 h treatment with 100 pM estrogen to induce proliferation (Figure 5A). Prior to evaluating proliferation, a live–dead staining was performed to confirm that treatment with fulvestrant did not result in diminished cellular viability (Figure S7). Cell proliferation was evaluated by *K*_i_-67, confirming that exposure to fulvestrant impairs proliferation (Figure 5B). A dose-dependent relationship was observed with the 50 nM dose resulting in intermediate levels of proliferation in the 3D spheroids when compared to nearly complete elimination of proliferation with the 100 nM dose (Figure 5C). Negative control with no fulvestrant and no estrogen exhibited basal levels of proliferation that were lower than the 50 nM treated spheroids supporting the need for E2 to induce proliferation in ER+ breast cancer (Figure 5C). A positive control with no drug and 100 pM estrogen demonstrated significantly high levels of proliferation, which is supported by the literature.^62^ Results obtained from a conventional two-dimensional (2D) drug study demonstrated a visible response starting at 15 nM fulvestrant treatment with a maximal response observed at concentrations of 25 nM and higher (Figure S8). The 2D studies verified that concentrations above 25 nM were necessary to halt proliferation; however, there was a clear difference in the cellular response between the 2D and 3D cell culture systems (Figure 5 and Figure S8). A direct comparison of E2-induced proliferation between the two systems found that a drug concentration of 15 nM in the 2D system was comparable to a 50 nM dose in the 3D system (Figure S9). The findings here support the concept that cells cultured in a 3D environment exhibit an altered response to endocrine therapies when compared to cells cultures in 2D.^6,40,63^ This observed change in endocrine response between 2D and 3D could be attributed to multiple factors including strong cell-to-cell interaction, compact cell packing, deposition of ECM between cells, or the existence of different cell layers within 3D tumor spheroid.^64^ Additionally, a previous study suggests that 3D tumor spheroids better recapitulate the tumor microenvironment (TME) by mimicking strong cell-to-cell interactions and mass transfer limitations of metabolites, oxygen, and drugs, which the 2D model of cancer cell culture fails to replicate.^6,13−15^ These findings support the importance of using 3D spheroids for drug screening due to the observed differences in cellular response between the 2D and 3D cell culture systems.

## Conclusions

The findings from this study highlight an alternative approach to generate 3D spheroids incorporating an easy-to-use, inexpensive scaffold that can be used for high-throughput spheroid generation and drug screening. The growth and viability of MCF7 spheroids were maintained for 7 days by continuous media flow generated using a customized gravity-driven media flow system to eliminate the need for syringe pumps. Additionally, since the number of cells in each droplet can be controlled easily, a study was carried out to determine the effect of the number of parent cells in each droplet on spheroid growth. Results suggest that a minimum of 10 or more encapsulated cells are needed to generate a growing spheroid while fewer than 10 parent cells produced stagnant 3D spheroids. A drug study was performed treating the spheroids with fulvestrant followed by interrogating the spheroids for proliferation in the presence of estrogen. Following fulvestrant exposure, the spheroids showed significantly less proliferation in the presence of estrogen, confirming an altered response to endocrine therapies in 3D cultured cells. In the future, this system can be used for on-chip interrogation and evaluation of different biological systems such as the interaction between different types of cells and their effect on drug resistance.

## Supporting Information Available

Discussions of chemicals, microfluidic wafer fabrication, gravity-driven media infusion system to support 3D cell growth, image analysis, COMSOL simulations to measure the velocity and the fluid shear stress profile, and 2D drug response analysis, figures of gravity-driven media infusion system, computational fluid dynamics, in-house built MATLAB image analysis code, spheroid generation in the 150 μm microfluidic trapping array, on-chip viability staining, spheroid growth in 150 μm trapping array when start with fewer than 10 encapsulated MCF-7 cells, cellular viability spheroids were treated with 100 nM fulvestrant, endocrine therapy response in 2D cell cultured, and altered response to endocrine therapy when ER^+^ breast cancer cultured in 2D compared to 3D, and table of different parameters to generate appropriate size droplets (PDF)
Movie of generation of hydrogel droplets in flow-focusing junction (AVI)
Movie of droplet trapping in trapping array (AVI)
Movie of media flushes out Novec 7500 oil from the device (AVI)

## Supplementary Material

ab2c00491_si_001.pdf

ab2c00491_si_002.avi

ab2c00491_si_003.avi

ab2c00491_si_004.avi
