# Supplementary material for: Generation of 3D Spheroids Using a Thiol–Acrylate Hydrogel Scaffold to Study Endocrine Response in ER+ Breast Cancer
Source: ACS Biomater Sci Eng. 2022 Aug 24;8(9):3977–85. doi: 10.1021/acsbiomaterials.2c00491 (PMC9472224; doi:10.1021/acsbiomaterials.2c00491)
Supplement: Supplementary file 1 — ab2c00491_si_001.pdf [file ab2c00491_si_001.pdf]

Supporting Information for

Generation of 3D spheroids using a thiol-acrylate hydrogel scaffold to study endocrine response in ER^+ breast cancer

Anowar H. Khan[1], Sophia P. Zhou[2], Margaret Moe[3], Braulio A. Ortega Quesada[3], Khashayar R. Bajgiran[3], Haley R. Lassiter[4], James A. Dorman[3], Elizabeth C. Martin[4], John A. Pojman[1] and Adam T. Melvin[3]

[1]*Department of Chemistry, Louisiana State University, Baton Rouge, LA, 70803*

[2]*Department of Bioengineering, Rice University, Houston, TX 77005*

[3]*Cain Department of Chemical Engineering, Louisiana State University, Baton Rouge, LA,70803*

[4]*Biological and Agricultural Engineering, Louisiana State University, Baton Rouge, LA, 70803*

Table of contents

Figure S1: Continuous gravity-driven media infusion setup to support 3D cell growth.

Figure S2: COMSOL Multiphysics 5.3 with the computational fluid dynamics (CFD) module

Figure S3: Bright field image analysis using in-house built MATLAB image analysis code.

Figure S4: Generation of ER^+ MCF-7 spheroids in the 150 μm microfluidic trapping array.

Figure S5: On-chip viability staining of MCF-7 spheroids generated in the 150 μm trapping array.

Figure S6: Fewer than 10 encapsulated MCF-7 cells resulted in poor spheroid growth in the 150 μm trapping array

Figure S7: Assessment of cellular viability in the 3D spheroids treated with 100 nM fulvestrant.

Figure S8: ER^+ breast cancer exhibits an altered response to endocrine therapy in 2D cultured cells

Figure S9: ER^+ breast cancer exhibits an altered response to endocrine therapy when cultured in 2D compared to 3D

Movie S1: Generation of hydrogel droplets in flow-focusing junction

Movie S2: Droplet trapping in trapping array

Movie S3: Media flushes out Novec 7500 oil from the device

Table S1: Different parameters for perfect size droplet generation that were used for spheroid generation

Chemicals

Polyethylene glycol diacrylate (PEGDA) (Mn 700), Estrogen (E2 or β-Estradiol), and Fulvestrant (ICI) were purchased from Sigma Aldrich. Ethoxylated trimethylolpropane tri(3-mercaptopropionate) 1300 (ETTMP 1300) was generously donated by Evans Chemetics LP. Aquapel (Model No: 98990A-6) was purchased from Amazon and 008-FluoroSurfactant was purchased from RAN biotechnologies. Triton 100x and Bovine albumin serum (BSA) were purchased from Life Science. 4% Paraformaldehyde (PFA) solution, Hoechst 33342 (nuclei stain), Calcein AM (live stain), and ethidium homodimer-1 (dead stain) were purchased from Thermo Fisher Scientific. Anti-Ki-67 Mouse Monoclonal Antibody (Alexa Fluor® 488) was purchased from VWR, DMSO was purchased Corning, and Novec 7500 was purchased from 3M. Extracellular buffer (ECB: 5.036 mM HEPES, 136.89 mM NaCl, 2.68 mM KCl, 2.066 mM $MgCl_2•6H_2O$, 1.8 mM $CaCl_2•2H_2O$, and 5.55 mM glucose) was used as the solvent for hydrogel synthesis. Unless otherwise stated, all other reagents were obtained from Sigma Aldrich.

Wafer fabrication

The device geometry was designed in AutoCAD (Autodesk, USA) to generate two transparency masks (CAD/Art). One of the masks contained the fluidic channel and the other mask contained the trapping array. The silicon master was fabricated by first depositing SU-8 2050 (Kayaku Advanced Materials), a negative photoresist polymer, onto a 4” silicon wafer (University Wafer) using a spin coater (WS-650MZ-23NPP, Laurell, USA) to generate a final channel height of 100 μm for the fluidic layer followed by baking at 65°C and 95°C for 15 min and 45 min, respectively. After the wafer was cooled to room temperature, the transparency mask containing fluidic channels was placed on top of the wafer, followed by exposure to UV light (1.4 mW/cm^2) for 55 s in a custom-built UV exposure set-up using a B100-AP lamp (VWR). The wafer was then baked at 65 °C for 15 min and 95 °C for 1 h after UV exposure and then allowed to cool to room temperature. Next, the second 300 μm trapping layer was fabricated using a two-step process. First, a 150 μm-thick layer of SU-8 2050 was deposited onto the wafer, followed by baking at 65°C and 95°C for 30 min and 90 min, respectively. Next, the second layer of SU-8 2050 (also 150 μm thick) was deposited on the same wafer, followed by baking at 65°C and 95°C for 30 min and 120 min. After the wafer was cooled at room temperature, the transparency mask for the trapping array (either the 150 or 300 μm diameter traps) was placed on top of the wafer, followed by exposure to UV light

(1.4 mW/cm^2) for 70 s in the same exposure system. The wafer was baked at 65 °C for 15 min and 95 °C for 2 h after UV exposure. After cooling at 25 °C for 30 min, SU-8 developer (Kayaku Advanced Materials) was used to remove all uncrosslinked SU-8. The wafer was hard-baked at 150 °C for 1.5 h to increase durability and then treated with a silane (tridecafluoro-1,1,2,2-tetrahydrooctyl trichlorosilane, Sigma-Aldrich) in a vacuum to deposit a thin layer on the surface of the wafer, which facilitates the detachment of the polymeric replicas.

Incorporation of continuous gravity-driven media infusion system to support 3D cell growth

A custom standalone media infusion system was designed to continuously supply the growing cells with culture media. It was constructed using a single test-tube holder to support both the media reservoir and waste collector at a height of 13 and 11.5 cm, respectively (Figure S1). A 5 mL BD plastic syringe with a 23-gauge needle was used for both the reservoir and collector which were connected to the device with Tygon tubing. The media reservoir was connected to the oil inlet port of the device while the waste reservoir was connected to the device outlet. To prevent any media leakage through the hydrogel inlet port, it was closed by connecting to a closed 5 mL syringe using Tygon tubing. To support cell growth, the media was replenished every 24 hours by adding 4 mL media into the media reservoir and at the same time removing the same volume from the waste collector. It was observed that the TA hydrogel started to degrade ~ 30 h after synthesis with complete degradation by 48 h. Due to this fact, the media was not replenished between 24 h to 48 h since it may result in some spheroid loss due to fluid motion.[1]

Image analysis

Brightfield image analysis was implemented using custom MATLAB code (R2021a) that is capable of processing batch-fed images in a folder and exporting all data into a Microsoft Excel file. The code was designed to convert the images into grayscale followed by filtering out noises and uses image contrast to locate and identify the spheroids within the traps (Figure S2). A mask was also created over the circular traps preventing them from being mistakenly identified as spheroids. Spheroids were detected as shapes since they are darker than the surrounding areas; thus, the area (A) and perimeter (P) of the spheroids were directly calculated using pixel area. This area was converted using the provided scale and exported into an Excel file for monitoring. The shape index (ShI) of spheroids was also tracked to determine which spheroids to use for analysis

since it determines how close the spheroids are to spherical geometry shapes. ShI was determined as follows;[2-4]

$$\mathrm{ShI} = \frac{4\pi A}{P^2} \qquad \text{Eqn. 1}$$

The ShI value of 0 indicates a straight line, while 1 represents a perfect sphere. While measuring the diameter and area of the spheroid, only spheroids possessing a shape index (ShI) value of 0.6 to 1 were considered for analysis. This is very important piece of information since it helps to differentiate between a loosely interacting cell aggregates when ShI value falls below 0.6 and tumor spheroid posing strong intra cellular interaction when ShI value is greater than 0.6.[2, 3] The diameter of the spheroid was estimated using an average of the height and width of the spheroids. The program has adjustable filters to maximize efficiency; the code was set to filter out spheroids with a diameter less than 40 μm and spheroids with an area less than 1300 μm^2. It was done because anything below 40 μm diameter contains too few cells to be considered to be spheroid according to some previously published work.[2, 3] After analyzing the images' different parameters (such as diameter, area, ShI), the results were automatically transferred into an Excel file. Later, those data were carefully analyzed using Origin software.

Fluorescence image analysis was done using ImageJ (NIH). First, the total fluorescence intensity of the spheroid was measured as integrated density. Afterward, the background intensity of a small spot near the spheroid was measured. Finally, the normalized fluorescence intensity (NFI) was calculated as the following equation,

$$\mathrm{NFI} = \{\text{integrated density} - (\text{area of the spheroid} * \text{fluorescence of background readings})\}$$

The NFI was measured for 50 similar sized spheroids from each set of experiments and compared afterward.

COMSOL simulations

COMSOL Multiphysics 5.3 with the computational fluid dynamics (CFD) module was used to model the profile velocity and the fluid shear stress (FSS) profile into the trapping array. Water fluidic settings were used since the viscosity and density of the cell media are very similar to water. The following parameters were specified: 300 μm for the diameter of the traps, 100 μm height of the fluidic channel, 300 μm for the height of the traps, and the velocity in the inlet was 5 μL/min

considering 4 mL media flow in 24 hours, and non-slip boundary conditions. The velocity profile and fluid shear stress were obtained for the given set-up.

Two-dimensional (2D) drug response and image analysis

The initial stock of fulvestrant (ICI-182780) and estrogen was made and serially diluted in DMSO and kept at -20 °C until further use. Stripped media (phenol free DMEM media containing 5% FBS charcoal dextran, 1% glutamax, 1% non-essential amino acid, 1% essential amino acid, 1% sodium pyruvate, and 1% penicillin-streptomycin) aliquots were generated with either fulvestrant (1, 2.5, 5, 10, 15, 25, 50 and 100 nM) or estrogen (100 pM) and stored at 4 °C. To study the effect of fulvestrant or estrogen on 2D MCF-7 proliferation, cells were grown in 96-well plates (Corning) for 72 hours in MCF-7 cell culture media, followed by 24 hours of culture in stripped media. For the drug study, the 2D MCF-7 cells were exposed to the 100 µL fulvestrant spiked stripped media for 9 hours. Afterward, the fulvestrant spiked media was removed from the wells and replaced with 100 µL of 17β-estradiol (E2 or estrogen) spiked stripped media. The estrogen-spiked media was replenished every 24 hours. A vehicle control experiment was performed the same using DMSO (2 µL DMSO per mL of stripped media) in lieu of fulvestrant. Terminal Proliferation (Ki-67, Bio Legend) and nuclei (Hoechst 33342, Thermo Scientific) staining were carried out as follows.

At the end of the experiment, cells were fixed by treating cells with 4% paraformaldehyde (PFA) solution for 30 minutes. Followed by washing with PBS three times, the samples were then permeabilized by treating with PBS containing 1 %(w/v) Triton X 100 for 8 hours. Afterward, cells were washed with a blocking buffer (0.5 % w/v BSA in PBS) for 30 minutes and followed by a PBS wash three times. Stain solution was made under a biosafety hood containing 4900 µL 0.25% BSA, 70 µL of Ki-67 stain (1:70 dilution), and 30 µL nuclear stain Hoechst 33342 (60 µM). Afterward, 100 µL of stain solution was added to each well and incubated overnight at room temperature in the dark. Samples were washed with PBS three times prior to the imaging. Cellular fluorescence was visualized using a Leica DMi8 inverted microscope outfitted with a FITC filter cube (Excitation 460 to 500 nm, Emission 512 to 542 nm), DAPI filter (Excitation 325 to 375 nm, Emission 435 to 485 nm), and brightfield applications at 20x objective. Digital images were acquired using the Flash 4.0 high-speed camera (Hamamatsu) with a fixed exposure time of 600 ms for the FITC filter (green, Ki-67 positive cells), 50 ms for DAPI (blue, nucleus), and 35 ms for

brightfield. Fluorescence image analysis was done using ImageJ (NIH) as mentioned earlier section. Normalized fluorescence intensity was measured for the area of approximate size 38,000 μm^2 and this area was kept constant in all data sets so that different data sets can be compared. Furthermore, this area was selected since for the 3D study the average size of the analyzed spheroid was ~38,000 μm^2. Additionally, for each drug concentration, the normalized fluorescent intensity of 50 spots was measured.

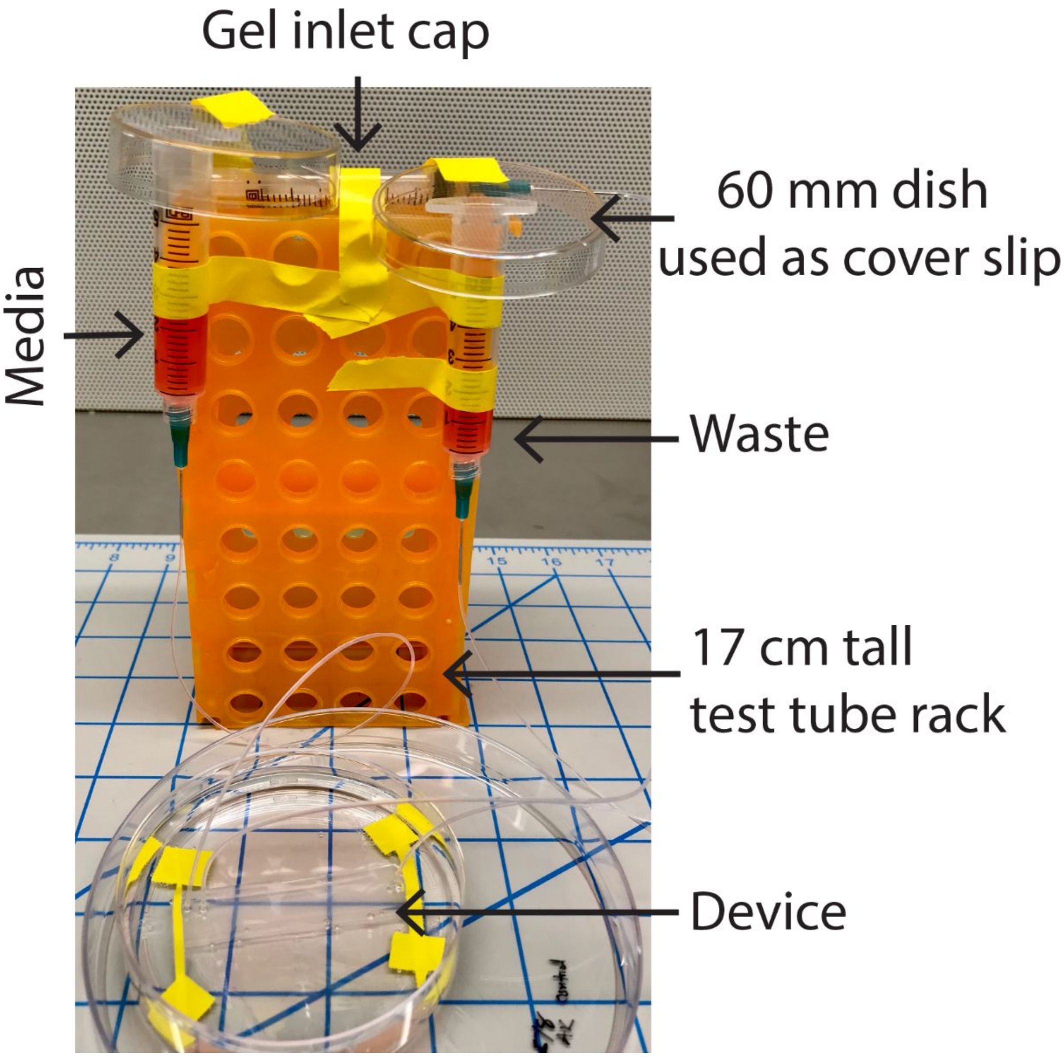

Figure S1. Continuous gravity-driven media infusion setup to support 3D cell growth. The gravity-driven system consisted of two BD plastic syringes (5.0 mL) connected to the one inlet port (media reservoir) and outlet port (waste collector) of the device. The media infusion syringe was positioned slightly higher (~1.5 cm) to induce flow. For cell culture purposes, media was replenished every 24 h by adding 4 mL culture media into the media reservoir and at the same time removing spent media from the waster collector. Another plastic syringe (1 or 3 mL) was used to close the gel inlet of the device.

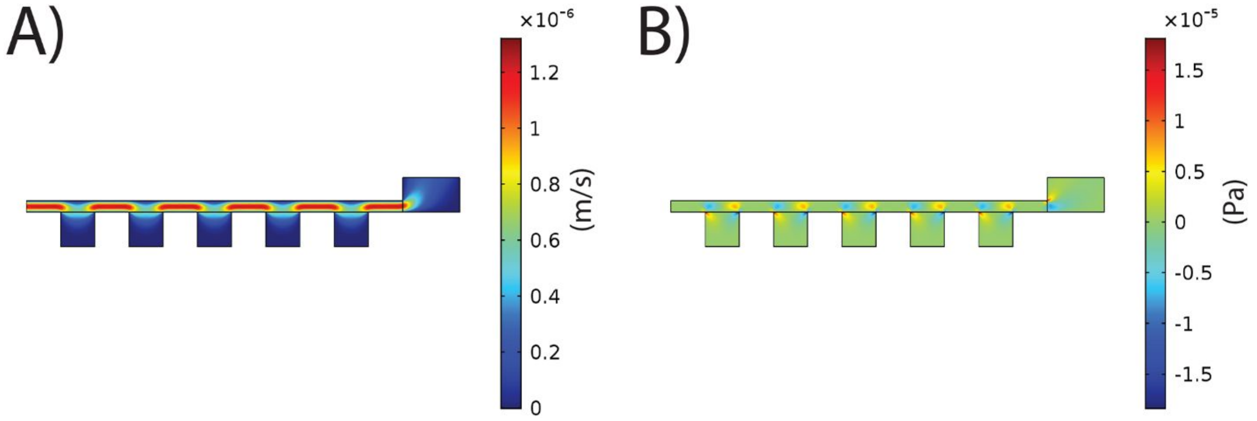

Figure S2: COMSOL Multiphysics 5.3 with the computational fluid dynamics (CFD) module was used to model A) the velocity profile and B) the fluid shear stress profile into the trapping array.

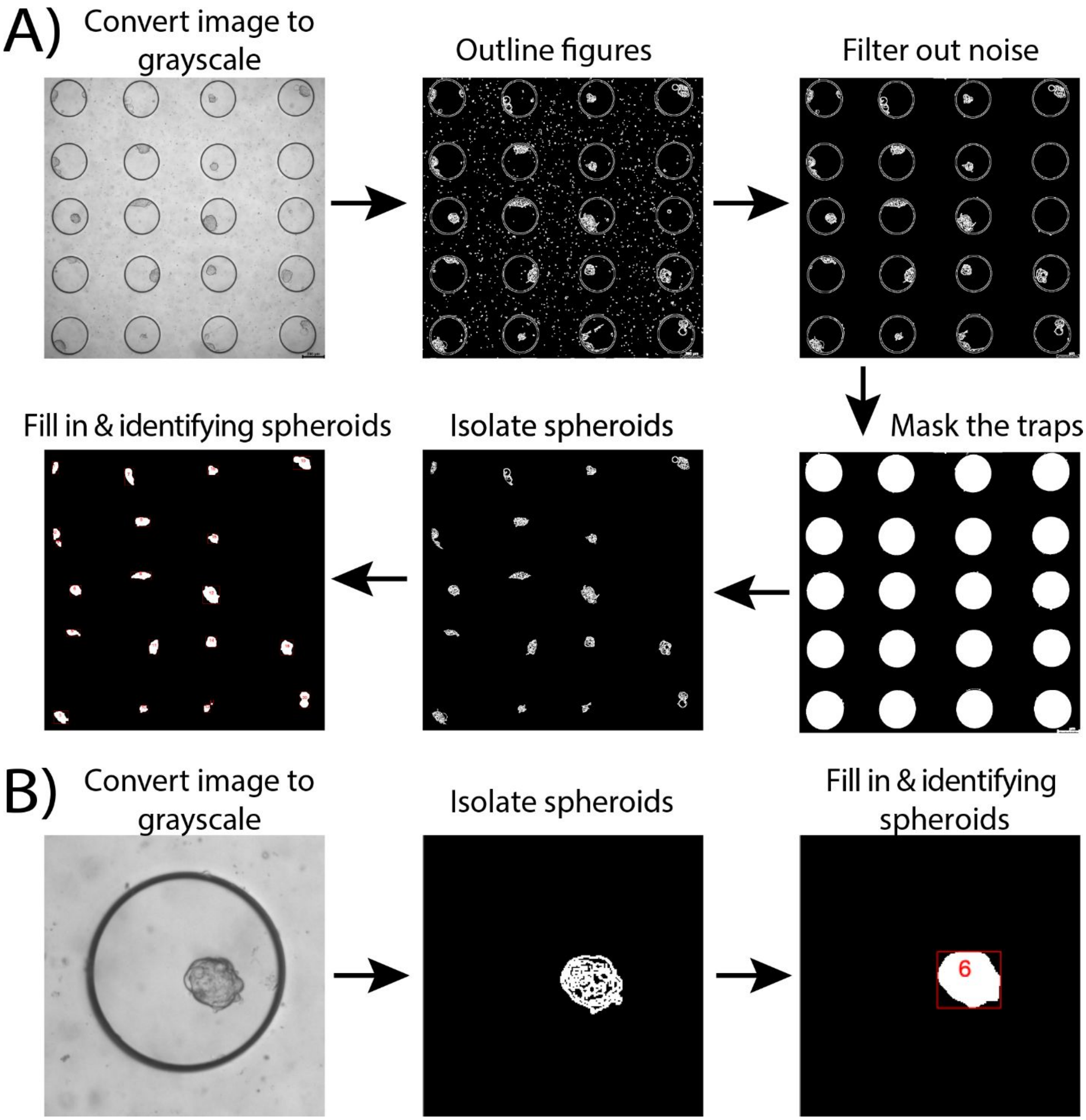

Figure S3. Bright field image analysis using in-house built MATLAB image analysis code. A) Six different steps were used for spheroid identification. B) Representative spheroid identification in a single trap occurs by first isolating the spheroid followed by filling in the image within the boundary to achieve a whole spheroid.

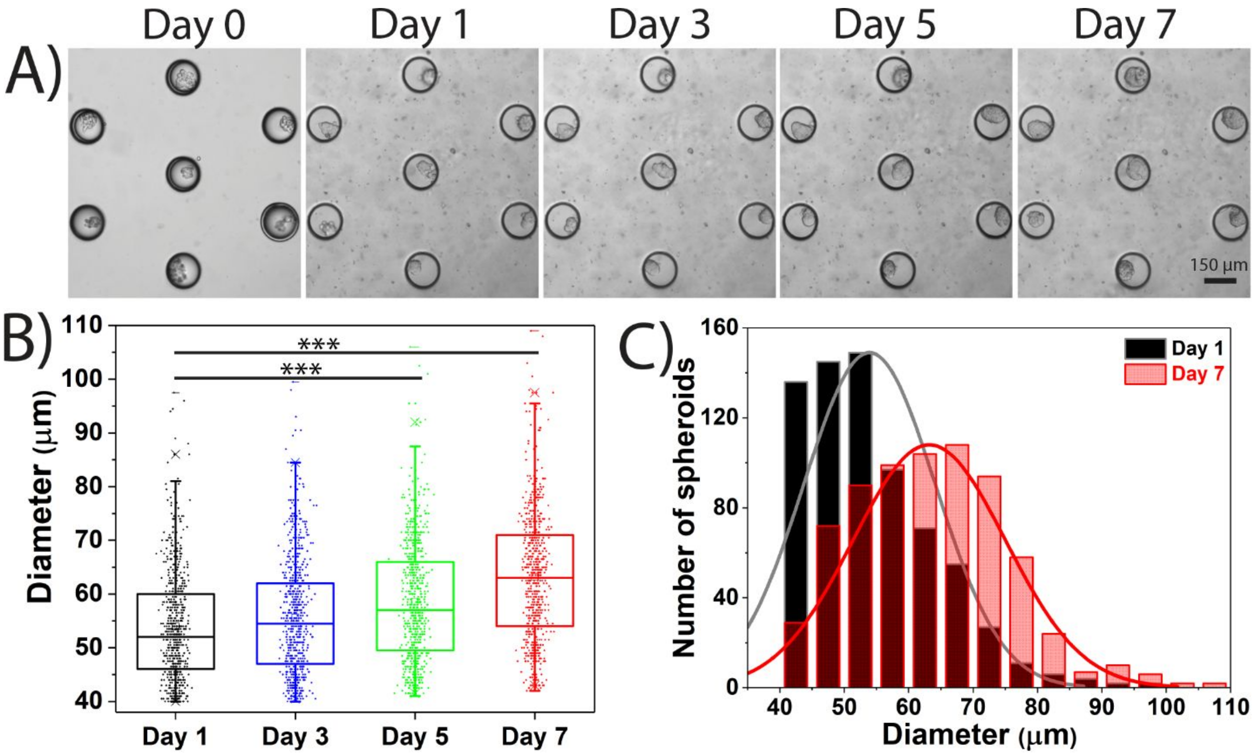

Figure S4. Generation of ER+ MCF-7 spheroids in the 150 µm microfluidic trapping array. A) Brightfield images of seven representative traps containing MCF-7 spheroids were collected at days 1, 3, 5, and 7. B) Calculated diameters of a population of 700 spheroids accomplished using a custom MATLAB algorithm confirms cell growth in the device. C) Size distribution of the generated spheroids on days 1 and 7 show a shift in spheroid size as a function of time. ***$p<0.001$

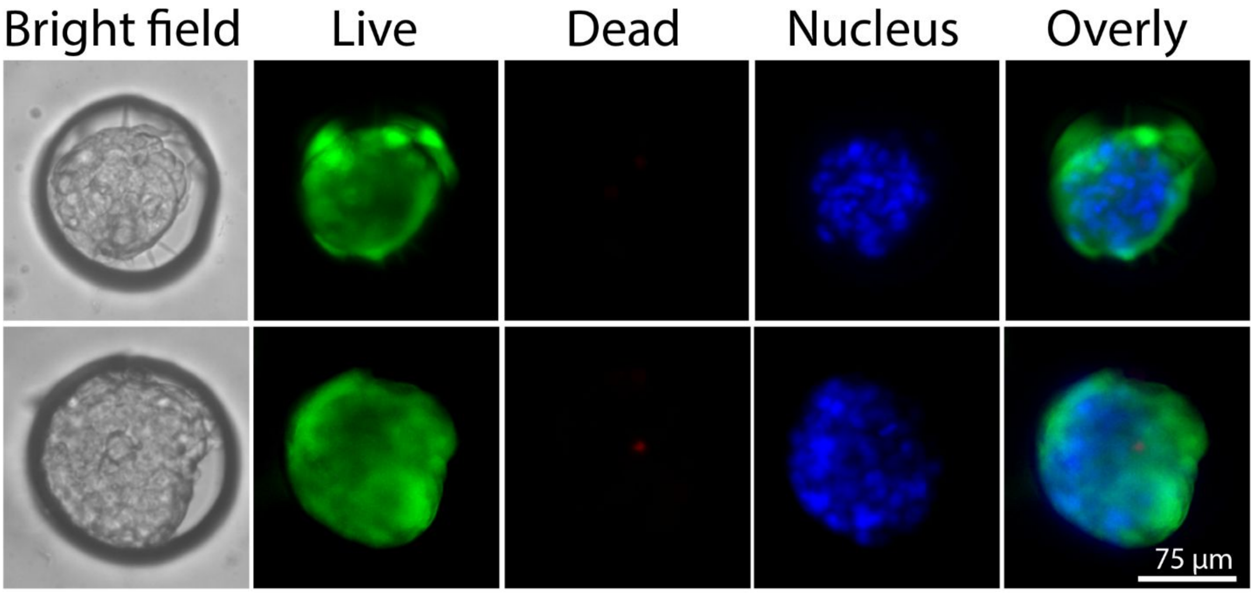

Figure S5. On-chip viability staining of MCF-7 spheroids generated in the 150 µm trapping array. After 7 days of culture, the spheroids were incubated with live and dead fluorescent stains. Representative images of three spheroids are shown for bright field, green (live, Calcein-AM), red (dead, Ethidium homodimer), blue (nuclei, Hoechst 33342), and an overlay image.

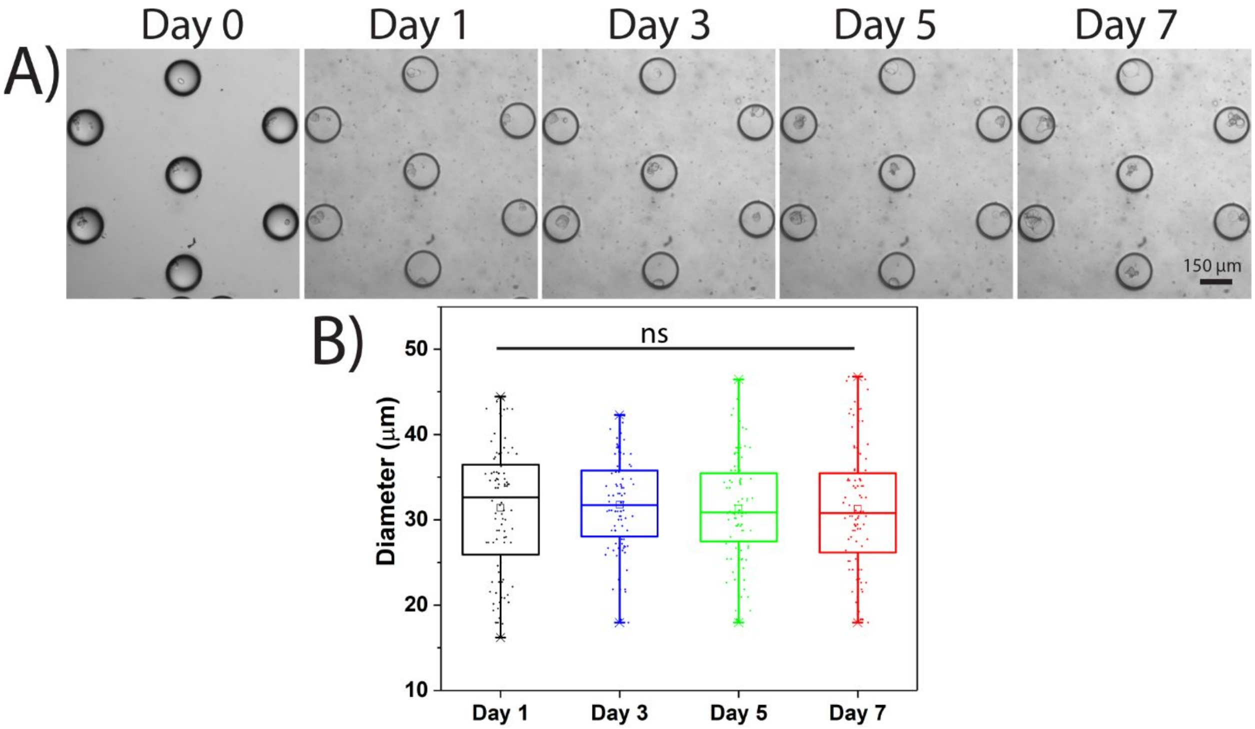

Figure S6. Fewer than 10 encapsulated MCF-7 cells resulted in poor spheroid growth in the 150 µm trapping array. A) Brightfield images of traps containing less than ten MCF-7 cells after encapsulation were taken at a different time interval. B) Measured diameter of 3D spheroids generated from less than 10 cells over time. (ns indicates statistically non-significant when $p>0.05$).

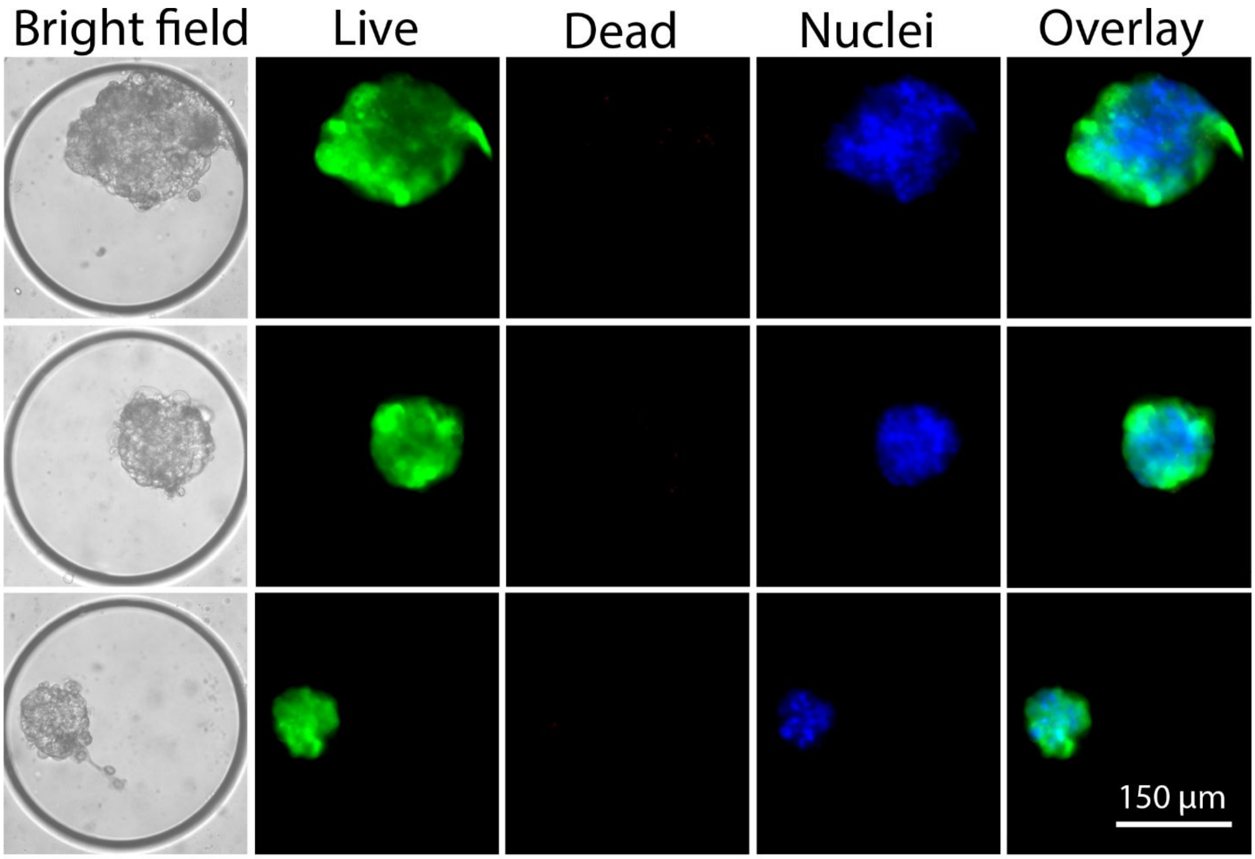

Figure S7. Assessment of cellular viability in the 3D spheroids treated with 100 nM fulvestrant. On-chip viability of MCF-7 spheroids which were exposed to 100 nM fulvestrant (ICI) for 9 hours followed by 48 hours of exposure to 100 pM estrogen (E2) containing striped media. Representative images are shown for brightfield, green indicates live cells (Calcine AM), red indicates dead cells (Ethidium homodimer), blue indicates the nucleus of the cell (Hoechst 33342) and an overlay image.

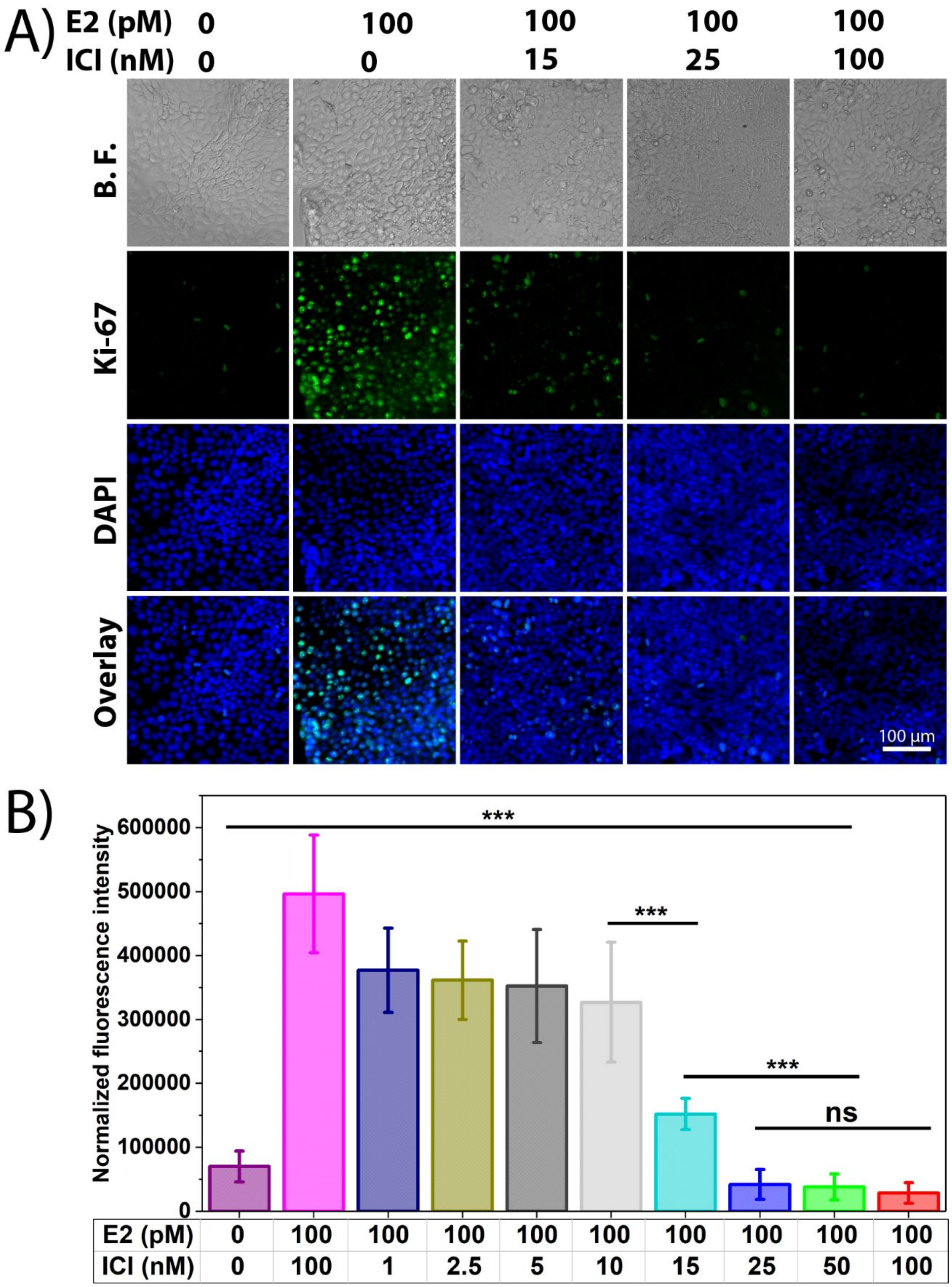

++

Figure S8. ER+ breast cancer exhibits an altered response to endocrine therapy in 2D cultured cells in the 96-well plate. A) Evaluation of cellular proliferation by Ki-67 induced by

estrogen (E2) in the presence or absence of fulvestrant (ICI) in 2D MCF-7 monolayer cells. B) Quantification of normalized fluorescence coupled with one-way ANOVA to demonstrate statistically significant changes in cellular proliferation due to drug treatments. (*** indicates statistically significant $p<0.0001$, ns indicates statistically non-significant $p>0.05$).

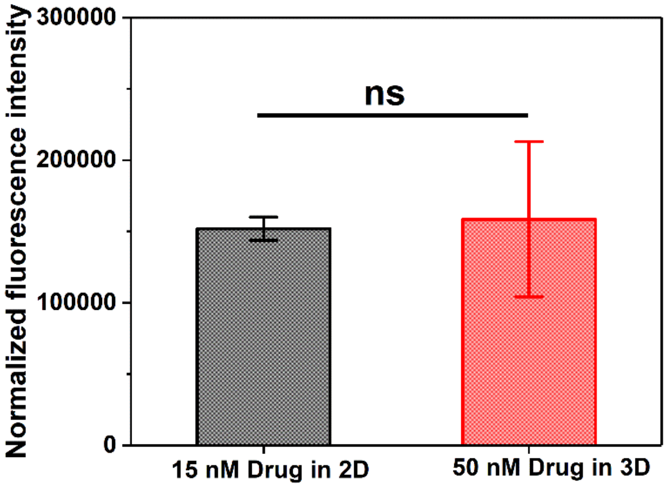

Figure S9. ER$^+$ breast cancer exhibits an altered response to endocrine therapy when cultured in 2D compared to 3D. Quantification of cellular proliferation by Ki-67 induced by estrogen (E2) in the presence of 15 nM fulvestrant (ICI) in 2D and 50 nM ICI in 3D environment demonstrated that the signals are statistically non-significant (obtained from one-way ANOVA analysis). This indicates that the 3D cell culture of MCF-7 requires a higher drug dose compared to 2D in order to obtain a comparable drug response. (ns indicates statistically non-significant $p>0.01$)

Table S1. Different parameters for perfect size droplet generation that were used for spheroid generation

Traps diameter (μm)	Cell seeding density (cells/mL hydrogel)	To make appropriate droplet size	
		Novec 7500 oil containing 0.5 % (w/w) surfactant flow rate (μL/h)	Aqueous prepolymer containing cells flow rate (μL/h)
150	8 x 10^6	750	190
300	5 x 10^6	230	600

Movie S1: Generation of hydrogel droplets in flow-focusing junction

Movie S2: Droplet trapping in trapping array

Movie S3: Media flushes out Novec 7500 oil from the device

References

1. Khan, A. H.; Cook, J. K.; Wortmann III, W. J.; Kersker, N. D.; Rao, A.; Pojman, J. A.; Melvin, A. T., *J. Biomed. Mater. Res. Part B Appl. Biomater.* **2020,** *108* (5), 2294-2307.
2. Sart, S.; Tomasi, R. F. X.; Amselem, G.; Baroud, C. N., *Nat. Commun.* **2017,** *8* (1), 469.
3. Lee, J. M.; Choi, J. W.; Ahrberg, C. D.; Choi, H. W.; Ha, J. H.; Mun, S. G.; Mo, S. J.; Chung, B. G., *Microsyst. Nanoeng.* **2020,** *6* (1), 52.
4. Amaral, R. L. F.; Miranda, M.; Marcato, P. D.; Swiech, K., *Frontiers in Physiology* **2017,** *8* (605).
